# (*E*)-2-[4-(Trifluoro­meth­oxy)benzyl­idene]indan-1-one

**DOI:** 10.1107/S1600536811028698

**Published:** 2011-07-30

**Authors:** Mohamed Ashraf Ali, Rusli Ismail, Soo Choon Tan, Mohd Mustaqim Rosli, Hoong-Kun Fun

**Affiliations:** a Institute for Research in Molecular Medicine, Universiti Sains Malaysia, 11800 USM, Penang, Malaysia; b X-ray Crystallography Unit, School of Physics, Universiti Sains Malaysia, 11800 USM, Penang, Malaysia

## Abstract

In the title compound, C_17_H_11_F_3_O_2_, the dihydro­indene ring is approximately planar with a maximum deviation of 0.024 (2) Å and makes a dihedral angle of 3.17 (8) Å with the adjacent benzene ring. In the crystal, mol­ecules are inter­connected by C—H⋯O inter­actions, forming an infinite chain along the *c* axis.

## Related literature

For the biological background to dihydro­indeno and heterocyclic derivatives, see: Dinges *et al.* (2006 ▶); Garton *et al.* (2006 ▶); Lin *et al.* (1997 ▶); Hsieh *et al.* (1998 ▶); Ko *et al.* (2003 ▶). For a related structure, see: Ali *et al.* (2011 ▶). For standard bond lengths, see: Allen *et al.* (1987 ▶) For the stability of the temperature controller used in the data collection, see: Cosier & Glazer (1986 ▶).
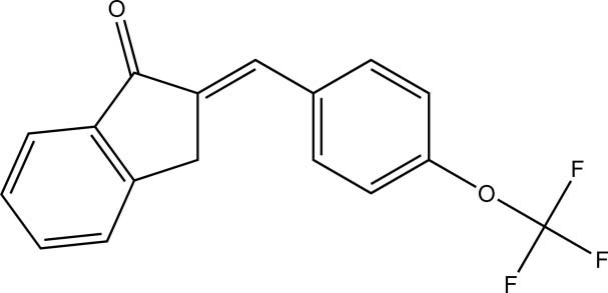


## Experimental

### 

#### Crystal data

C_17_H_11_F_3_O_2_
*M* *_r_* = 304.26
Monoclinic, 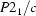

*a* = 15.2216 (4) Å
*b* = 14.6734 (4) Å
*c* = 6.1463 (1) Å
β = 95.872 (1)°
*V* = 1365.59 (6) Å^3^
*Z* = 4
Mo *K*α radiation
μ = 0.12 mm^−1^
*T* = 100 K
0.38 × 0.20 × 0.18 mm

#### Data collection

Bruker SMART APEXII CCD area-detector diffractometer
Absorption correction: multi-scan (*SADABS*; Bruker, 2009 ▶) *T*_min_ = 0.955, *T*_max_ = 0.978
27814 measured reflections
4043 independent reflections
3389 reflections with *I* > 2σ(*I*)
*R*_int_ = 0.036

#### Refinement

*R*[*F* ^2^ > 2σ(*F* ^2^)] = 0.069
*wR*(*F* ^2^) = 0.181
*S* = 1.11
4043 reflections
199 parameters
H-atom parameters constrained
Δρ_max_ = 0.87 e Å^−3^
Δρ_min_ = −0.28 e Å^−3^

### 

Data collection: *APEX2* (Bruker, 2009 ▶); cell refinement: *SAINT* (Bruker, 2009 ▶); data reduction: *SAINT*; program(s) used to solve structure: *SHELXTL* (Sheldrick, 2008 ▶); program(s) used to refine structure: *SHELXTL*; molecular graphics: *SHELXTL*; software used to prepare material for publication: *SHELXTL* and *PLATON* (Spek, 2009 ▶).

## Supplementary Material

Crystal structure: contains datablock(s) I, global. DOI: 10.1107/S1600536811028698/sj5181sup1.cif

Structure factors: contains datablock(s) I. DOI: 10.1107/S1600536811028698/sj5181Isup2.hkl

Additional supplementary materials:  crystallographic information; 3D view; checkCIF report

## Figures and Tables

**Table 1:** Hydrogen-bond geometry (Å, °)

*D*—H⋯*A*	*D*—H	H⋯*A*	*D*⋯*A*	*D*—H⋯*A*
C1—H1*B*⋯O1^i^	0.99	2.51	3.304 (2)	137
C10—H10*A*⋯O1^ii^	0.95	2.45	3.309 (2)	151

Symmetry codes: (i) 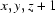; (ii) 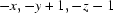.

**Fig. 1.**
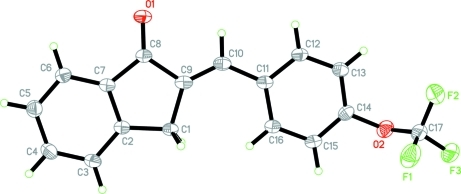
The molecular structure, showing 50% probability displacement ellipsoids. Hydrogen atoms are shown as spheres of arbitrary radius.

**Fig. 2.**
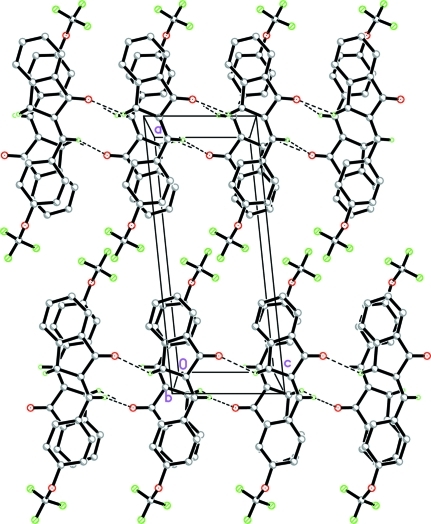
The packing of (I) showing infinite chains along c axis. Dashed lines indicate hydrogen bonds. H atoms not involved in the hydrogen bond interactions have been omitted for clarity.

## supplementary crystallographic information

### Comment

Recently it has been reported that dihydroindeno derivatives represent a novel class of KDR kinase inhibitors (Dinges *et al.* 2006) with inhibition of both KDR and cKit in the appropriate tumor types. They also have the potential to produce antitumor effects through two distinct mechanisms. Inhibition of cKit should result in direct effects on the tumor cell phenotype, while inhibition of KDR should produce indirect effects *via* disruption of endothelial cell function (Garton *et al.* 2006). Some of the heterocyclic derivatives inhibited the release of chemical mediators from mast cells, neutrophils, macrophages, and microglial cells *in vitro*, and suppressed the oedematous response *in vivo* (Lin *et al.* 1997, Hsieh *et al.* 1998, Ko *et al.* 2003). Many antitumor drugs have been developed for prostate cancer patients but their intolerable systemic toxicity often limits their clinical use. The title compound contains the dihydroindene unit and its structure is reported here, Fig 1.

All bond lengths (Allen *et al.*, 1987) and angles in (I) are within normal ranges. The dihydroindene ring is planar with a maximum deviation of 0.024 (2)Å and it makes a dihedral angle of 3.17 (8)° with the adjancent benzene ring (Fig. 1). In the crystal, the molecules are interconnected by C—H···O interactions (Table 1) to form infinite chains along the *c* axis (Fig. 2).

### Experimental

A mixture of 2,3-dihydro-1*H*-indene-1-one (0.001 mmol) and 4-trifluoromethoxy benzaldehyde (0.001 mmol) was dissolved in methanol (10 ml) and 30% sodium hydroxide solution (5 ml) was added and stirred for 5 h. After completion of the reaction as evident from TLC, the mixture was poured into crushed ice and then neutralized with Con HCl. The precipitated solid was filtered, washed with water and recrystallized from ethanol to reveal the title compound as light yellow crystals.

### Refinement

All H-atoms were positioned geometrically and refined using a riding model, with C—H = 0.95 and 0.99 Å, and with *U*_iso_ = 1.2*U*_eq_(C).

### Crystal data

**Table:**

C_17_H_11_F_3_O_2_	*F*(000) = 624
*M**_r_* = 304.26	*D*_x_ = 1.480 Mg m^−^^3^
Monoclinic, *P*2_1_/*c*	Mo *K*α radiation, λ = 0.71073 Å
Hall symbol: -P 2ybc	Cell parameters from 9932 reflections
*a* = 15.2216 (4) Å	θ = 2.7–30.0°
*b* = 14.6734 (4) Å	µ = 0.12 mm^−^^1^
*c* = 6.1463 (1) Å	*T* = 100 K
β = 95.872 (1)°	Block, light-yellow
*V* = 1365.59 (6) Å^3^	0.38 × 0.20 × 0.18 mm
*Z* = 4	

### Data collection

**Table:**

Bruker SMART APEXII CCD area-detector diffractometer	4043 independent reflections
Radiation source: fine-focus sealed tube	3389 reflections with *I* > 2σ(*I*)
graphite	*R*_int_ = 0.036
φ and ω scans	θ_max_ = 30.2°, θ_min_ = 1.3°
Absorption correction: multi-scan (*SADABS*; Bruker, 2009)	*h* = −21→21
*T*_min_ = 0.955, *T*_max_ = 0.978	*k* = −19→20
27814 measured reflections	*l* = −8→8

### Refinement

**Table:**

Refinement on *F*^2^	Primary atom site location: structure-invariant direct methods
Least-squares matrix: full	Secondary atom site location: difference Fourier map
*R*[*F*^2^ > 2σ(*F*^2^)] = 0.069	Hydrogen site location: inferred from neighbouring sites
*wR*(*F*^2^) = 0.181	H-atom parameters constrained
*S* = 1.11	*w* = 1/[σ^2^(*F*_o_^2^) + (0.0634*P*)^2^ + 1.8592*P*] where *P* = (*F*_o_^2^ + 2*F*_c_^2^)/3
4043 reflections	(Δ/σ)_max_ < 0.001
199 parameters	Δρ_max_ = 0.87 e Å^−^^3^
0 restraints	Δρ_min_ = −0.28 e Å^−^^3^

### Special details

**Table:**

Experimental. The crystal was placed in the cold stream of an Oxford Cryosystems Cobra open-flow nitrogen cryostat (Cosier & Glazer, 1986) operating at 100.0 (1) K.
Geometry. All e.s.d.'s (except the e.s.d. in the dihedral angle between two l.s. planes) are estimated using the full covariance matrix. The cell e.s.d.'s are taken into account individually in the estimation of e.s.d.'s in distances, angles and torsion angles; correlations between e.s.d.'s in cell parameters are only used when they are defined by crystal symmetry. An approximate (isotropic) treatment of cell e.s.d.'s is used for estimating e.s.d.'s involving l.s. planes.
Refinement. Refinement of *F*^2^ against ALL reflections. The weighted *R*-factor *wR* and goodness of fit *S* are based on *F*^2^, conventional *R*-factors *R* are based on *F*, with *F* set to zero for negative *F*^2^. The threshold expression of *F*^2^ > σ(*F*^2^) is used only for calculating *R*-factors(gt) *etc*. and is not relevant to the choice of reflections for refinement. *R*-factors based on *F*^2^ are statistically about twice as large as those based on *F*, and *R*- factors based on ALL data will be even larger.

### Fractional atomic coordinates and isotropic or equivalent isotropic displacement parameters (Å^2^)

**Table:**

	*x*	*y*	*z*	*U*_iso_*/*U*_eq_	
F1	0.40870 (11)	0.43026 (14)	0.4881 (3)	0.0493 (5)	
F2	0.47465 (10)	0.43584 (13)	0.1983 (3)	0.0481 (4)	
F3	0.51278 (10)	0.33503 (13)	0.4426 (3)	0.0467 (4)	
O1	−0.10593 (11)	0.45341 (11)	−0.4450 (2)	0.0277 (3)	
O2	0.38837 (11)	0.31658 (12)	0.2471 (3)	0.0315 (4)	
C1	−0.06329 (13)	0.35420 (13)	0.0949 (3)	0.0211 (4)	
H1A	−0.0435	0.2906	0.1220	0.025*	
H1B	−0.0424	0.3919	0.2234	0.025*	
C2	−0.16251 (14)	0.35906 (13)	0.0484 (3)	0.0205 (4)	
C3	−0.22701 (14)	0.33207 (14)	0.1806 (3)	0.0245 (4)	
H3A	−0.2111	0.3077	0.3224	0.029*	
C4	−0.31497 (15)	0.34167 (16)	0.1000 (4)	0.0294 (5)	
H4A	−0.3595	0.3233	0.1883	0.035*	
C5	−0.33995 (15)	0.37768 (16)	−0.1085 (4)	0.0287 (4)	
H5A	−0.4007	0.3834	−0.1596	0.034*	
C6	−0.27604 (14)	0.40491 (14)	−0.2400 (3)	0.0244 (4)	
H6A	−0.2921	0.4295	−0.3815	0.029*	
C7	−0.18764 (13)	0.39517 (13)	−0.1592 (3)	0.0199 (4)	
C8	−0.10851 (14)	0.41903 (13)	−0.2650 (3)	0.0217 (4)	
C9	−0.02974 (15)	0.39174 (14)	−0.1129 (3)	0.0236 (4)	
C10	0.05182 (15)	0.40510 (14)	−0.1712 (3)	0.0238 (4)	
H10A	0.0541	0.4300	−0.3132	0.029*	
C11	0.13779 (14)	0.38722 (14)	−0.0497 (3)	0.0231 (4)	
C12	0.21341 (14)	0.40795 (14)	−0.1522 (3)	0.0229 (4)	
H12A	0.2071	0.4368	−0.2913	0.027*	
C13	0.29726 (14)	0.38732 (14)	−0.0553 (3)	0.0245 (4)	
H13A	0.3481	0.3999	−0.1280	0.029*	
C14	0.30495 (14)	0.34785 (14)	0.1510 (3)	0.0231 (4)	
C15	0.23247 (14)	0.32961 (14)	0.2623 (3)	0.0232 (4)	
H15A	0.2398	0.3041	0.4051	0.028*	
C16	0.14908 (14)	0.34919 (14)	0.1619 (3)	0.0247 (4)	
H16A	0.0987	0.3368	0.2366	0.030*	
C17	0.44420 (15)	0.37880 (19)	0.3410 (4)	0.0334 (5)	

### Atomic displacement parameters (Å^2^)

**Table:**

	*U*^11^	*U*^22^	*U*^33^	*U*^12^	*U*^13^	*U*^23^
F1	0.0378 (9)	0.0719 (12)	0.0369 (8)	0.0042 (8)	−0.0022 (6)	−0.0208 (8)
F2	0.0344 (8)	0.0604 (11)	0.0489 (9)	−0.0086 (7)	0.0013 (7)	0.0168 (8)
F3	0.0275 (7)	0.0728 (12)	0.0387 (8)	0.0091 (7)	−0.0019 (6)	0.0124 (8)
O1	0.0325 (8)	0.0316 (8)	0.0196 (7)	−0.0001 (6)	0.0050 (6)	0.0053 (6)
O2	0.0285 (8)	0.0346 (9)	0.0309 (8)	0.0073 (7)	0.0000 (6)	0.0027 (6)
C1	0.0264 (10)	0.0186 (9)	0.0186 (8)	−0.0006 (7)	0.0030 (7)	0.0032 (6)
C2	0.0271 (10)	0.0157 (8)	0.0188 (8)	0.0003 (7)	0.0039 (7)	−0.0003 (6)
C3	0.0305 (11)	0.0230 (9)	0.0211 (9)	0.0009 (8)	0.0076 (7)	0.0024 (7)
C4	0.0296 (11)	0.0299 (11)	0.0302 (11)	0.0011 (9)	0.0110 (8)	0.0025 (8)
C5	0.0262 (10)	0.0294 (11)	0.0306 (11)	0.0015 (8)	0.0035 (8)	0.0004 (8)
C6	0.0276 (10)	0.0238 (9)	0.0218 (9)	0.0018 (8)	0.0019 (7)	0.0002 (7)
C7	0.0261 (10)	0.0160 (8)	0.0178 (8)	−0.0006 (7)	0.0042 (7)	−0.0007 (6)
C8	0.0271 (10)	0.0192 (9)	0.0192 (8)	0.0002 (7)	0.0037 (7)	0.0014 (6)
C9	0.0331 (11)	0.0198 (9)	0.0183 (8)	0.0003 (8)	0.0040 (7)	0.0004 (7)
C10	0.0327 (11)	0.0208 (9)	0.0184 (8)	−0.0006 (8)	0.0043 (7)	−0.0009 (7)
C11	0.0285 (10)	0.0216 (9)	0.0194 (9)	−0.0007 (8)	0.0029 (7)	0.0024 (7)
C12	0.0300 (10)	0.0213 (9)	0.0173 (8)	−0.0030 (8)	0.0023 (7)	0.0006 (7)
C13	0.0281 (10)	0.0252 (10)	0.0208 (9)	0.0004 (8)	0.0050 (7)	−0.0010 (7)
C14	0.0271 (10)	0.0201 (9)	0.0214 (9)	0.0029 (7)	−0.0013 (7)	−0.0009 (7)
C15	0.0310 (10)	0.0207 (9)	0.0175 (8)	0.0004 (8)	0.0011 (7)	0.0014 (7)
C16	0.0272 (10)	0.0257 (10)	0.0214 (9)	−0.0010 (8)	0.0036 (7)	0.0057 (7)
C17	0.0248 (11)	0.0483 (14)	0.0268 (10)	0.0028 (10)	0.0014 (8)	0.0033 (9)

### Geometric parameters (Å, °)

**Table:**

F1—C17	1.334 (3)	C6—C7	1.393 (3)
F2—C17	1.330 (3)	C6—H6A	0.9500
F3—C17	1.326 (3)	C7—C8	1.469 (3)
O1—C8	1.220 (2)	C8—C9	1.497 (3)
O2—C17	1.337 (3)	C9—C10	1.341 (3)
O2—C14	1.421 (2)	C10—C11	1.463 (3)
C1—C2	1.510 (3)	C10—H10A	0.9500
C1—C9	1.526 (3)	C11—C12	1.401 (3)
C1—H1A	0.9900	C11—C16	1.409 (3)
C1—H1B	0.9900	C12—C13	1.386 (3)
C2—C3	1.395 (3)	C12—H12A	0.9500
C2—C7	1.398 (3)	C13—C14	1.388 (3)
C3—C4	1.387 (3)	C13—H13A	0.9500
C3—H3A	0.9500	C14—C15	1.382 (3)
C4—C5	1.402 (3)	C15—C16	1.384 (3)
C4—H4A	0.9500	C15—H15A	0.9500
C5—C6	1.386 (3)	C16—H16A	0.9500
C5—H5A	0.9500		
			
C17—O2—C14	117.40 (18)	C10—C9—C1	132.40 (19)
C2—C1—C9	103.78 (16)	C8—C9—C1	107.68 (17)
C2—C1—H1A	111.0	C9—C10—C11	129.92 (19)
C9—C1—H1A	111.0	C9—C10—H10A	115.0
C2—C1—H1B	111.0	C11—C10—H10A	115.0
C9—C1—H1B	111.0	C12—C11—C16	118.21 (19)
H1A—C1—H1B	109.0	C12—C11—C10	117.70 (17)
C3—C2—C7	119.75 (19)	C16—C11—C10	124.08 (19)
C3—C2—C1	128.79 (18)	C13—C12—C11	121.48 (18)
C7—C2—C1	111.46 (17)	C13—C12—H12A	119.3
C4—C3—C2	118.33 (19)	C11—C12—H12A	119.3
C4—C3—H3A	120.8	C12—C13—C14	118.15 (19)
C2—C3—H3A	120.8	C12—C13—H13A	120.9
C3—C4—C5	121.8 (2)	C14—C13—H13A	120.9
C3—C4—H4A	119.1	C15—C14—C13	122.39 (19)
C5—C4—H4A	119.1	C15—C14—O2	117.17 (18)
C6—C5—C4	120.1 (2)	C13—C14—O2	120.18 (19)
C6—C5—H5A	120.0	C14—C15—C16	118.77 (18)
C4—C5—H5A	120.0	C14—C15—H15A	120.6
C5—C6—C7	118.19 (19)	C16—C15—H15A	120.6
C5—C6—H6A	120.9	C15—C16—C11	120.89 (19)
C7—C6—H6A	120.9	C15—C16—H16A	119.6
C6—C7—C2	121.90 (18)	C11—C16—H16A	119.6
C6—C7—C8	128.58 (18)	F3—C17—F2	107.74 (19)
C2—C7—C8	109.52 (17)	F3—C17—F1	108.00 (19)
O1—C8—C7	127.16 (19)	F2—C17—F1	106.5 (2)
O1—C8—C9	125.34 (19)	F3—C17—O2	107.9 (2)
C7—C8—C9	107.49 (16)	F2—C17—O2	113.2 (2)
C10—C9—C8	119.89 (18)	F1—C17—O2	113.3 (2)
			
C9—C1—C2—C3	178.8 (2)	C2—C1—C9—C10	179.7 (2)
C9—C1—C2—C7	−0.3 (2)	C2—C1—C9—C8	1.8 (2)
C7—C2—C3—C4	0.4 (3)	C8—C9—C10—C11	177.73 (19)
C1—C2—C3—C4	−178.7 (2)	C1—C9—C10—C11	0.1 (4)
C2—C3—C4—C5	−0.2 (3)	C9—C10—C11—C12	179.9 (2)
C3—C4—C5—C6	−0.1 (4)	C9—C10—C11—C16	1.0 (4)
C4—C5—C6—C7	0.1 (3)	C16—C11—C12—C13	3.6 (3)
C5—C6—C7—C2	0.0 (3)	C10—C11—C12—C13	−175.43 (19)
C5—C6—C7—C8	−179.7 (2)	C11—C12—C13—C14	−2.0 (3)
C3—C2—C7—C6	−0.3 (3)	C12—C13—C14—C15	−0.7 (3)
C1—C2—C7—C6	178.95 (18)	C12—C13—C14—O2	173.25 (18)
C3—C2—C7—C8	179.50 (18)	C17—O2—C14—C15	−105.6 (2)
C1—C2—C7—C8	−1.3 (2)	C17—O2—C14—C13	80.1 (3)
C6—C7—C8—O1	1.1 (3)	C13—C14—C15—C16	1.8 (3)
C2—C7—C8—O1	−178.7 (2)	O2—C14—C15—C16	−172.35 (18)
C6—C7—C8—C9	−177.8 (2)	C14—C15—C16—C11	−0.2 (3)
C2—C7—C8—C9	2.4 (2)	C12—C11—C16—C15	−2.4 (3)
O1—C8—C9—C10	0.3 (3)	C10—C11—C16—C15	176.49 (19)
C7—C8—C9—C10	179.24 (18)	C14—O2—C17—F3	172.57 (17)
O1—C8—C9—C1	178.51 (19)	C14—O2—C17—F2	−68.3 (3)
C7—C8—C9—C1	−2.6 (2)	C14—O2—C17—F1	53.1 (3)

### Hydrogen-bond geometry (Å, °)

**Table:**

*D*—H···*A*	*D*—H	H···*A*	*D*···*A*	*D*—H···*A*
C1—H1B···O1^i^	0.99	2.51	3.304 (2)	137
C10—H10A···O1^ii^	0.95	2.45	3.309 (2)	151

Symmetry codes: (i) *x*, *y*, *z*+1; (ii) −*x*, −*y*+1, −*z*−1.
